# Seasonal Metabolic Profiling and Anti-Inflammatory Potential of *Spatholobus suberectus* Leaves Based on Metabolomics and Network Pharmacology

**DOI:** 10.3390/plants15101509

**Published:** 2026-05-15

**Authors:** Meimei Luo, Dandan Yang, Shunda Jiang, Baoling Chen, Mei Yang, Yuanyuan Xu

**Affiliations:** 1 Guangxi Colleges and Universities Key Laboratory for Cultivation and Utilization of Subtropical Forest Plantation, School of Forestry, Guangxi University, Nanning 530004, China; 2309302014@st.gxu.edu.cn (M.L.);; 2 Guangxi Key Laboratory of Forest Ecology and Conservation, School of Forestry, Guangxi University, Nanning 530004, China; 3 Guangxi Zhuang Autonomous Region State-Owned Qipo Forest Farm, Nanning 530225, China; 4 Guangxi Zhuang Autonomous Region Forestry Science Research Institute, Nanning 530002, China

**Keywords:** *Spatholobus suberectus* leaves, untargeted metabolomics, seasonal variation, network pharmacology, anti-inflammatory activity

## Abstract

*Spatholobus suberectus* is a medicinal and edible plant widely recognized for its pharmacological potential. Although its stems have been extensively studied and utilized, its leaves are often discarded as agricultural waste, leading to significant resource underutilization. To promote the sustainable valorization of these leaves, this study aimed to provide a predictive evaluation of their bioactive constituents and pharmacological potential. Leaves of *S. suberectus* were collected at six growth stages (January, March, May, July, September and November). A total of 6750 metabolites were identified, primarily comprising amino acids and derivatives (26.74%), organic acids (15.33%), and bioactive secondary metabolites, including flavonoids and phenolic acids (27.98%). Metabolic profiling revealed clear seasonal patterns, allowing the classification of the six harvest months into three distinct stages: January and March (G1), May and September (G2), and July and November (G3). Among these, the G1 stage was notably enriched in defensive secondary metabolites, particularly flavonoids and phenolic acids. To predict the bioactivity of these metabolites and elucidate potential mechanisms of action, network pharmacology and molecular docking analyses were employed. Network pharmacology and molecular docking were employed to predict anti-inflammatory mechanisms. From the metabolome, 83 potential bioactive compounds were screened, interacting with 306 targets. Network analysis identified 60 core anti-inflammatory targets (e.g., TNF, AKT1, PTGS2, STAT3) that were significantly enriched in MAPK and PI3K-Akt pathways. Molecular docking revealed strong binding affinities, with pelargonidin showing the highest affinity for PTGS2 (−11.72 kcal/mol). Candidate metabolites peaked in January, and extracts from this period exhibited notable COX-2 inhibitory activity (IC50 = 16.41 μg/mL). This research provides essential chemical characterization and preliminary bioactivity evidence to support the valorization of *S. suberectus* leaves and identifies January as the optimal harvest time to maximize their bioactive potential.

## 1. Introduction

*Spatholobus suberectus* (*S. suberectus*), a medicinal plant belonging to the Fabaceae family, has been widely used in traditional Chinese medicine, particularly for its dried stems, which are rich in bioactive compounds such as flavonoids, phenolic acids, and terpenoids. These constituents exhibit multiple pharmacological activities, including the promotion of blood circulation [1,2], anticancer effects [3], antioxidant [4], and anti-inflammatory properties [5]. In South Asia, *S. suberectus* is commonly used as a food additive in products such as wines, teas, and soups [6]. However, while the stems have been widely studied, the leaves are often discarded during harvest, leading to significant resource waste. Developing the medicinal potential of these non-traditional parts could enable efficient whole-plant utilization. Plant leaves are often rich in flavonoids, phenolic acids, and sterols and are recognized for their strong antioxidant and anti-inflammatory activities [7,8,9]. Importantly, leaf harvesting is non-destructive to the plant, offering a more sustainable strategy. This approach has been validated by successful precedents: *Ginkgo biloba* leaves, rich in flavonol glycosides and terpene lactones, are now widely used in dietary supplements [10]; olive leaves are valued for polyphenols such as oleuropein and hydroxytyrosol [11]; and ginseng leaves exhibit unique pharmacological properties distinct from those of ginseng roots [12]. Inspired by these precedents and the reported traditional use of *S. suberectus* leaves for treating dermatitis, we hypothesize that these leaves possess a distinctive and valuable phytochemical profile. This study aims to move beyond general analogies by specifically characterizing these metabolites and evaluating their potential pharmacological significance relative to the traditionally used stems.

The composition and abundance of plant secondary metabolites are profoundly influenced by developmental stages and environmental fluctuations, making the selection of optimal harvest time critical for obtaining bioactive compounds. Previous studies have shown that the volatile oil components in *Garcinia* leaves peak in winter, with significant anti-inflammatory and cytotoxic activities [13], while phenolic and terpenoid compounds exhibit marked seasonal variations [14,15]. Such findings provide a scientific basis for precise harvesting and efficient utilization of medicinal plants. However, the seasonal dynamics of bioactive secondary metabolites in *S. suberectus* leaves remain largely unexplored. Traditional Chinese folk practices suggest that these leaves contain naturally occurring anti-inflammatory constituents, as they have been used externally to relieve neurodermatitis [16]. Inflammation is central to immune-related disorders, and natural products derived from anti-inflammatory plants are increasingly recognized as promising therapeutic agents [17].

In recent years, network pharmacology has emerged as a powerful tool for deciphering the mechanisms of multi-component herbal medicines by integrating systems biology and network analysis, thereby elucidating the multitarget interactions between bioactive compounds and disease-related pathways [18,19]. This approach has been successfully applied to predict antioxidant and anti-inflammatory mechanisms in various medicinal plants [20,21,22]. Additionally, molecular docking has become a key technique for validating the binding affinity between active compounds and core targets, providing structural insights into potential mechanisms of action [23]. Recognizing the limitations of single-method approaches, recent studies have increasingly integrated metabolomics with network pharmacology and molecular docking to link chemical profiles directly to pharmacological effects [24,25]. For instance, this integrated strategy has successfully revealed antioxidant components in wild jujube fruits [26] and red wines [27], elucidated anti-inflammatory mechanisms in *Cinnamomum cassia* essential oil [28], identified anti-obesity metabolites in fermented brown rice [29], and pinpointed betaine as a key anti-inflammatory and antioxidant component in drought-stressed maize roots targeting PPARG, PTGS2, and CASP3 [30]. However, no such integrated study has been applied to *S. suberectus* leaves, particularly regarding seasonal metabolite dynamics and anti-inflammatory potential.

Despite the extensive medicinal use of *S. suberectus* vine stems, its leaves are often discarded as agricultural waste, and the dynamic seasonal fluctuations in their chemical composition and anti-inflammatory potential remain largely unexplored. This raises critical scientific questions: How do the metabolic profiles of *S. suberectus* leaves change across different developmental stages? Which harvest time yields the highest accumulation of anti-inflammatory bioactive compounds? What are the potential molecular mechanisms and key targets through which these compounds exert their effects? To address these gaps, this research aims to systematically profile the metabolic patterns of *S. suberectus* leaves across six developmental stages using untargeted metabolomics. By integrating these findings with network pharmacology and molecular docking, we seek to identify how harvesting time influences the accumulation of bioactive compounds and to provide preliminary insights into their anti-inflammatory molecular mechanisms. The primary objectives are to suggest an optimal harvesting period and establish a scientific basis for the sustainable development and enhanced utilization of *S. suberectus* leaves in functional food and pharmaceutical applications.

## 2. Results

### 2.1. Metabolome Analysis

#### 2.1.1. Metabolomic Profiling of *S. suberectus* Leaves

This study involved the systematic metabolomic analysis of *S. suberectus* leaf samples collected at six time points using UPLC-MS/MS technology. A total of 6750 metabolites were identified, spanning a variety of functional categories such as amino acids, organic acids, flavonoids, alkaloids and phenolic acids (Table S1).

The most abundant group was amino acids and their derivatives, accounting for 26.74% of the total metabolome and comprising 1805 identified metabolites. The second most abundant category was organic acids, with 1035 metabolites (15.33%). The leaf samples also contained a diverse array of plant secondary metabolites, including benzenoids (754; 11.17%), flavonoids (371; 5.5%), phenolic acids (217; 3.21%), alkaloids (275; 4.07%), terpenoids (166; 2.46%), and lignans and coumarins (106; 1.57%). Together, these secondary metabolites represented over 25% of the total compounds identified (Figure S1).

**Figure 1:**
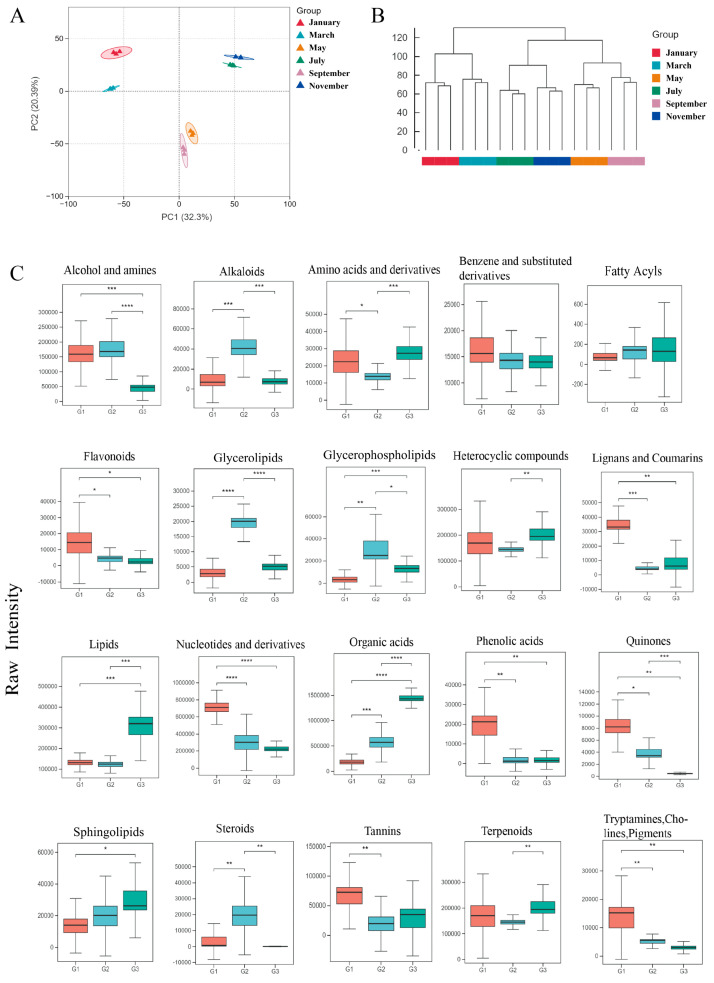
Metabolomic profiling and dynamic accumulation of metabolites across six stages. (**A**) Principal component analysis (PCA) of metabolites in six stages. (**B**) Hierarchical cluster analysis (HCA) of metabolites in six stages. (**C**) Raw intensity of different metabolite categories in different periods. The number of asterisks indicates the *p*-value: *p* < 0.05, one asterisk (*); *p* < 0.01, two asterisks (**); *p* < 0.001, three asterisks (***); *p* < 0.0001, four asterisks (****).

To investigate dynamic changes in the metabolite profiles of *S. suberectus* leaves at different sampling time points, we first performed principal component analysis (PCA) to reduce dimensionality and visualize sample distributions. The PCA results revealed clear clustering patterns among the six sampling stages, with biological replicates at each time point tightly clustered together, which indicates good data reproducibility. Together, the first two principal components (PC1 and PC2) accounted for 52.69% of the total variance (Figure 1A). The high quality of the data was demonstrated by the tight distribution of QC samples in the PCA plot (Figure S2). To better visualize the spatial separation of samples, a 3D PCA score plot incorporating PC3 was created; PC3 contributed an additional 8.71%, bringing the cumulative explanation to 61.40% (Figure S3). The PCA score plot showed that the samples could be divided into three distinct groups: G1 (January and March), G2 (May and September), and G3 (July and November). This suggests that there are significant temporal variations in metabolite composition. To validate the PCA results and further explore metabolic similarities, an unsupervised hierarchical cluster analysis (HCA) was conducted (Figure 1B). The HCA dendrogram was consistent with the PCA findings, supporting the classification of the six sampling stages into three groups: G1, G2 and G3.

The raw intensity of the 20 main metabolite categories was visualized using box plots (Figure 1C). The results revealed significant variations in metabolite accumulation patterns across the three groups, emphasizing the substantial impact of sampling time on metabolic profiles. Group G1 exhibited a notable increase in several bioactive secondary metabolite classes, including flavonoids, phenolic acids, lignans, coumarins, tannins, quinones and tryptamines. Flavonoids and phenolic acids were particularly enriched at this stage. In contrast, group G2 exhibited higher relative levels of glycerolipids, glycerophospholipids, steroids and alkaloids—metabolites associated with structural functions. Group G3 was characterized by a substantial increase in primary metabolites, such as amino acids and derivatives, organic acids, and lipids.

#### 2.1.2. Differential Metabolite Profiling and KEGG Enrichment in Different Periods

Based on the three metabolite groups (G1, G2 and G3) that were previously defined through PCA and HCA analyses, an orthogonal partial least squares discriminant analysis (OPLS-DA) was performed to compare each group in pairs and to identify DAMs (Figure S4A–C). To validate the reliability of the OPLS-DA models and ensure there was no overfitting, 200-time permutation tests were performed. The results confirmed the robustness of the models used for identifying differentially abundant metabolites (DAMs) (Figure S5). DAMs were selected based on VIP > 1, fold change ≥ 2 or ≤0.5 and *p*-value < 0.05 and then visualized using volcano plots. The results revealed 2326 significant DAMs between G1 and G2, including 1121 downregulated and 1205 upregulated metabolites (Figure S4D). A total of 2835 DAMs were detected between G3 and G1, of which 1831 were downregulated and 1004 were upregulated (Figure S4E). Meanwhile, the comparison between G3 and G2 identified 2527 DAMs, of which 1734 were downregulated and 493 were upregulated (Figure S4F).

**Figure 2:**
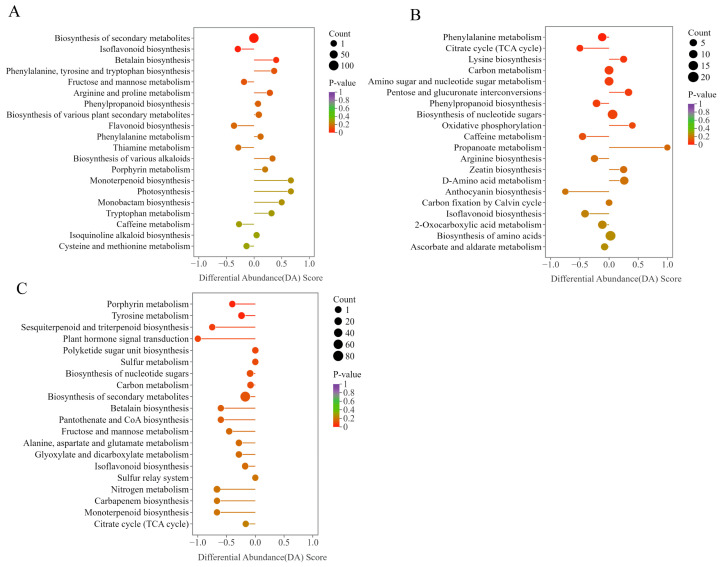
Pairwise comparisons between KEGG annotation and enrichment of differentially expressed metabolites at different periods. The DA score indicates the overall trend in a pathway: positive values represent upregulation, while negative values represent downregulation. (**A**) G2 vs. G1; (**B**) G3 vs. G1; (**C**) G3 vs. G2.

Further KEGG pathway enrichment analysis of the metabolomic data from different time points in *S. suberectus* leaves revealed dynamic metabolic transitions and functional shifts, as reflected by Differential Abundance (DA) scores representing overall pathway-level fluctuations. In the G2 vs. G1 comparison, isoflavonoid and flavonoid biosynthesis pathways showed a downregulated trend, while pathways involved in energy metabolism and cofactor biosynthesis, including those related to phenylalanine, tyrosine, tryptophan, arginine, and proline, were found to be upregulated (Figure 2A). In the G3 vs. G1 comparison, pathways related to growth and energy supply, such as lysine biosynthesis, pentose and glucuronate interconversions, and oxidative phosphorylation, were found to be upregulated. In contrast, defense-related secondary metabolic pathways, including the citrate cycle (TCA cycle), phenylalanine metabolism, and phenylpropanoid biosynthesis, were found to be downregulated (Figure 2B). Several key metabolic pathways exhibited a downward trend in the G3 vs. G2 comparison, including porphyrin metabolism, tyrosine metabolism, plant hormone signal transduction, and sesquiterpenoid and triterpenoid biosynthesis. These pathways cover primary metabolism, signal regulation, and defense-related secondary metabolism (Figure 2C).

### 2.2. Network Pharmacology Analysis

#### 2.2.1. Active Ingredient and Core Target Predictions

To identify potential anti-inflammatory metabolites in *S. suberectus* leaves, this study integrated pathway enrichment analysis of DAMs across different sampling time points with literature-based evidence regarding the biological functions of metabolites, their mechanisms of action in plants, and reported anti-inflammatory activities. Particular attention was given to plant metabolic pathways involved in inflammation regulation. Consequently, 14 inflammation-related metabolic pathways involving 116 DAMs were identified (Table S2).

Based on pharmacokinetic and drug-likeness evaluation using the SwissADME platform, a total of 91 potential bioactive compounds were selected from the 116 DAMs (Table S3). SwissTargetPrediction was then used to predict the molecular targets of these compounds, of which 83 successfully matched known targets. These included 17 phenolic acids (20.48%), 16 flavonoids (19.28%), 15 organic acids (18.07%), 14 amino acids and derivatives (16.87%), 12 alkaloids (14.46%), six lignans and coumarins (7.23%) and three others (3.61%) (Figure S6). Only targets with a prediction probability greater than zero were retained. After merging and deduplication, a total of 695 compound-associated targets were obtained (Table S4). To identify inflammation-related disease targets, the keyword “inflammation” was used to query the OMIM, DisGeNET and GeneCards databases. This yielded 1162 targets from OMIM, 134 from DisGeNET and 2000 inflammation-related targets from GeneCards. After merging the results and removing duplicates, 2527 unique inflammation-related disease targets were compiled (Table S5). The intersection of these 2527 disease targets and the 695 compound targets produced 306 shared targets (Figure S7).

**Figure 3:**
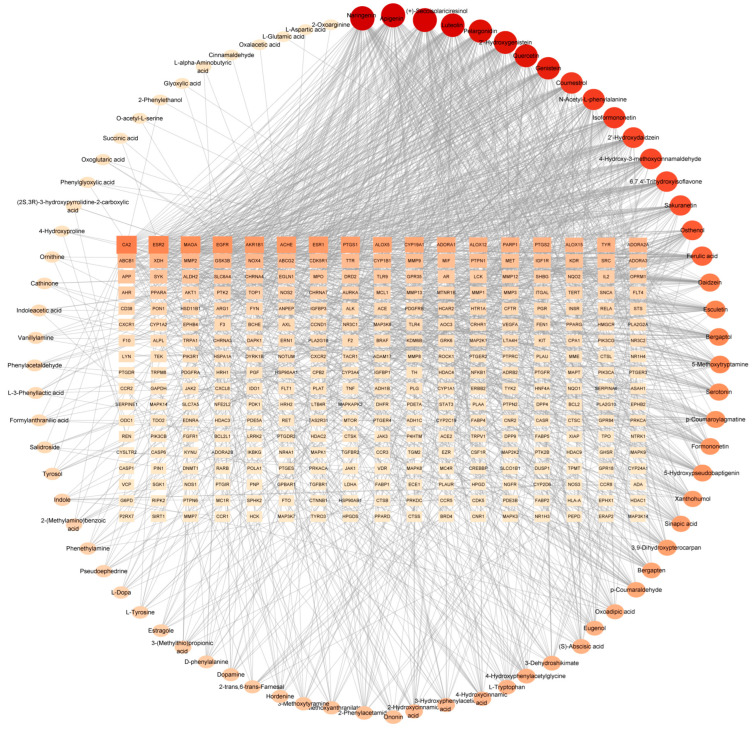
The “ingredient–target–disease” network of anti-inflammatory effects in *S. suberectus* leaves.

The 83 predicted active compounds and 306 intersecting targets were imported into Cytoscape 3.10.3 to construct and visualize the “ingredients–target–disease” network (Figure 3). The top 10 core compounds, ranked by degree value, included flavonoids such as naringenin (58), apigenin (57), luteolin (55), quercetin (52), genistein (50), 2′-hydroxygenistein (52), coumestrol (46), and pelargonidin (53); the lignan (+)-secoisolariciresinol (56); and the alanine derivative N-acetyl-L-phenylalanine (45).

A protein–protein interaction (PPI) analysis was performed on the 306 common targets using the STRING database (confidence score ≥ 0.700). This produced a network comprising 306 nodes and 5956 edges, visualized using Cytoscape software. Network topology analysis was then conducted using CentiScaPe 2.2, with the core targets being screened based on threshold values defined by median parameters (betweenness centrality ≥ 317.46, closeness centrality ≥ 0.00163, and degree ≥ 38.93). This ultimately identified 60 key targets (Figure S8). The top ten hub targets, ranked by degree, were: TNF (degree = 200), GAPDH (187), AKT1 (180), SRC (160), EGFR (159), STAT3 (150), BCL2 (139), PTGS2 (139), CTNNB1 (138), and NFKB1 (138).

#### 2.2.2. Results of GO Functional Enrichment and KEGG Pathway Analysis of Common Targets

**Figure 4:**
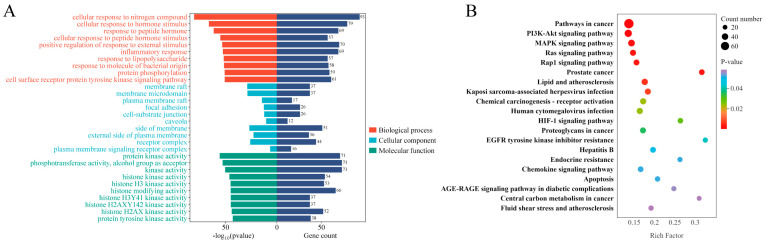
Functional annotation and pathway enrichment analysis of the common targets. (**A**) GO enrichment analysis of common targets. (**B**) KEGG pathway enrichment analysis of common targets.

Gene Ontology (GO) enrichment analysis of the 306 overlapping targets was performed using the Metascape platform with a significance threshold of *p* < 0.01. The top 10 terms from each GO category were visualized. The results revealed that the potential anti-inflammatory targets of *S. suberectus* leaf metabolites were mainly involved in Biological Processes such as inflammatory response, response to lipopolysaccharide, response to molecules of bacterial origin, cellular response to hormone stimulus, and positive regulation of response to external stimulus. The enriched Cellular Components included membrane raft, membrane microdomain, receptor complex, external side of plasma membrane, and focal adhesion. In terms of Molecular Functions, significant enrichment was observed in protein kinase activity, protein tyrosine kinase activity, and histone modifying activity (Figure 4A).

KEGG pathway enrichment analysis was conducted on the 306 overlapping targets using Metascape, setting the threshold at *p* < 0.01. Visualization of the top 20 pathways ranked by *p*-value revealed that *S. suberectus* leaf metabolite anti-inflammatory targets were significantly enriched in several classical inflammation-related signaling pathways, including the MAPK, PI3K-Akt, Ras, Rap1, lipid and atherosclerosis, and Kaposi sarcoma-associated herpesvirus infection pathways (Figure 4B).

#### 2.2.3. Results of Molecular Docking Analysis

**Figure 5:**
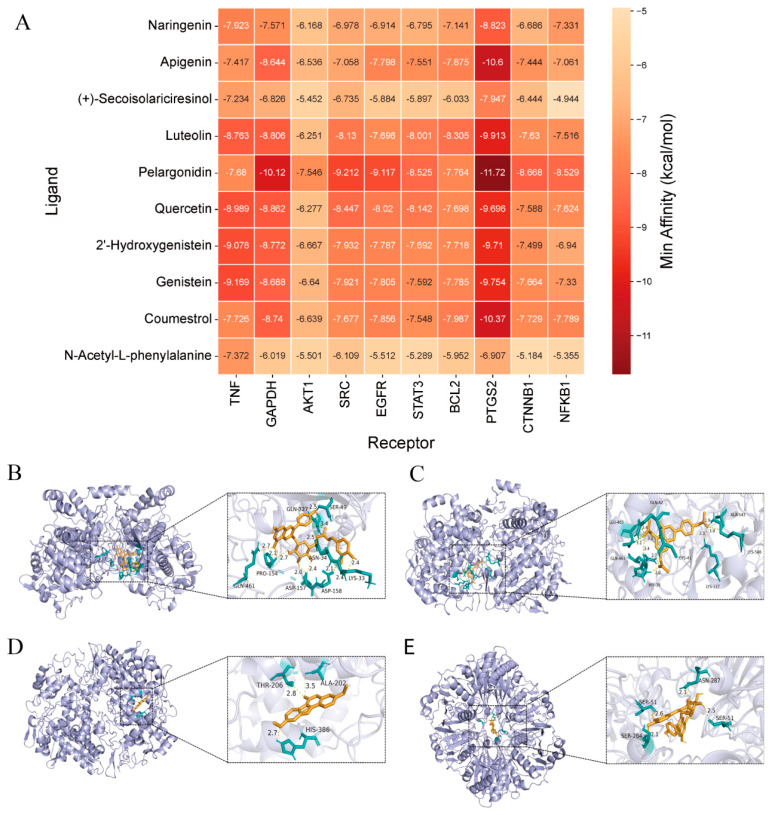
Molecular docking analysis of core active ingredients with key target proteins. (**A**) The docking results of core active ingredients and key target molecules. (**B**–**E**) Top 4 molecular docking results: (**B**) PTGS2–pelargonidin. (**C**) PTGS2–apigenin. (**D**) PTGS2–coumestrol. (**E**) GAPDH–pelargonidin.

To validate the predicted interactions between key active compounds and core targets, molecular docking was performed using AutoDock Vina. The top 10 key active compounds of *S. suberectus* with the highest degree values in the “compound–target–disease” network were selected as ligands, and the top 10 targets with the highest degree values from the PPI network were used as receptors. The results showed that all ligand–receptor binding energies were below −4.25 kcal/mol (100%), meeting the basic requirement for molecular binding (Figure 5A). Notably, 71% of the docking pairs (71 pairs) exhibited binding energies lower than −7.0 kcal/mol, suggesting strong binding activity between nearly two-thirds of the molecules.

The strongest binding affinity was observed between pelargonidin and PTGS2, with a binding energy of −11.72 kcal/mol. Flavonoids such as pelargonidin, apigenin, genistein, luteolin, quercetin, and coumestrol showed strong binding affinities to multiple targets. Among the targets, PTGS2 stood out as a core target, with 90% of the compounds displaying binding energies lower than −7.0 kcal/mol, and the top three docking interactions all centered on PTGS2. Additionally, TNF and GAPDH also showed strong binding affinities, with four and three compounds, respectively, displaying binding energies below −8.0 kcal/mol.

The four ligand–receptor pairs with the highest binding affinities were further analyzed by visualizing the docking conformations using PyMOL 2.6, with a focus on hydrogen bond interactions and spatial complementarity to reveal potential mechanisms of action (Figure 5B–E). Pelargonidin formed multiple stable hydrogen bonds with PTGS2, involving residues SER-49, GLN-327, ASN-34, PRO-154, LYS-33, GLN-461, ASP-157, and ASP-158. This extensive hydrogen bonding network, with bond lengths ranging from 2.1 to 3.2 Å, indicates a highly stable interaction. Other high-affinity ligands, such as apigenin and coumestrol, also formed multiple hydrogen bonds with their respective targets, further supporting the predicted target interactions from network pharmacology analysis. These findings provide a theoretical basis for subsequent experimental validation of biological activity. However, it should be noted that these docking scores serve as theoretical indicators of molecular binding potential rather than a definitive ranking of biological potency. While the low binding energies suggest strong affinity, the actual biological efficacy and pharmacodynamics of these metabolites remain to be confirmed through further in vitro and in vivo experimental validation.

### 2.3. In Vitro Validation of Antioxidant and Anti-Inflammatory Activities

#### 2.3.1. Temporal Variation in Anti-Inflammatory Compounds and Optimal Harvest Time Determination

A hierarchical clustering heatmap was constructed to analyze the temporal variation in the abundance of the ten most anti-inflammatory active compounds in *S. suberectus* leaves at different time points (Figure S9). The results showed that G1 enriched seven anti-inflammatory compounds that exhibited relatively high abundance and metabolic activity. Notably, the January sample showed a comparable number of anti-inflammatory compounds to the March sample but exhibited significantly higher overall abundance, indicating stronger functional metabolic advantages. Taking into account the quantity and abundance levels of anti-inflammatory compounds, January is recommended as the optimal harvest period for *S. suberectus* leaves to maximize their anti-inflammatory potential.

#### 2.3.2. DPPH and ABTS Radical Scavenging Activity

**Figure 6:**
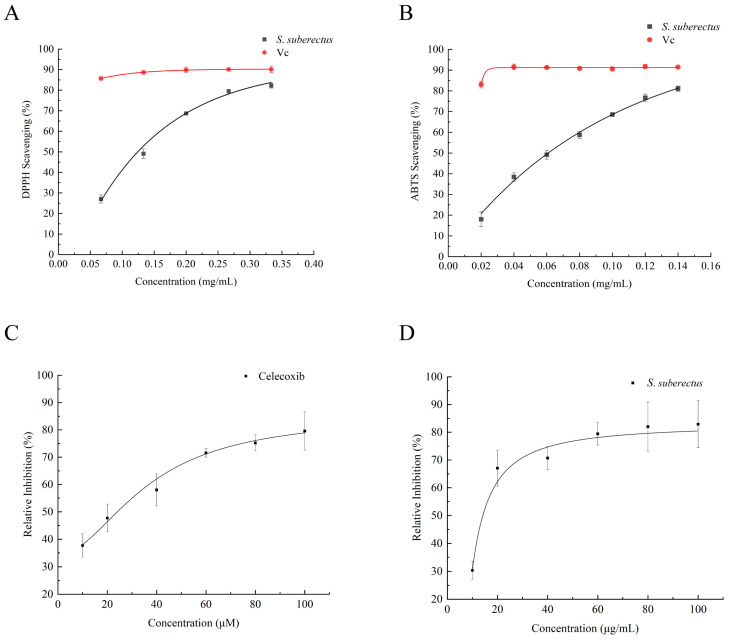
In vitro antioxidant and anti-inflammatory activities of the *S. suberectus* leaf extract. (**A**) Statistical chart showing the DPPH clearance effect of the crude extract from *S. suberectus* leaves at different concentrations. (**B**) Statistical chart showing the ABTS clearance effect of the crude extract from *S. suberectus* leaves at different concentrations. (**C**) Statistical chart showing the inhibitory effect of different concentrations of Celecoxib on COX-2. (**D**) Statistical chart showing the inhibitory effect of different concentrations of the crude extract from *S. suberectus* leaves on COX-2.

In vitro antioxidant assays were conducted on the samples collected in January to evaluate the antioxidant capacity of *S. suberectus* leaf extracts, using DPPH and ABTS radical scavenging methods with vitamin C (Vc) as the positive control. The results demonstrated clear concentration-dependent antioxidant activity, with IC_50_ values of 0.133 mg/mL (95% CI: 0.125–0.141) for DPPH and 0.062 mg/mL (95% CI: 0.058–0.066) for ABTS. At a concentration of 0.33 mg/mL, the DPPH radical scavenging rate was 82.25%, compared to 90.10% for vitamin C (Figure 6A). At a concentration of 0.14 mg/mL, the ABTS radical scavenging rate was 81.09% (Figure 6B). While this was slightly lower than that of vitamin C, the extract still exhibited strong electron-donating capacity and notable antioxidant potential at higher concentrations. To ensure accuracy, all assays were performed in triplicate (biological and technical replicates), and the final solvent concentration was maintained at <0.1% (*v*/*v*) to minimize interference.

#### 2.3.3. COX-2 Enzyme Inhibition Assay

Based on previous molecular docking results, which predicted that multiple compounds in the leaf extract would bind effectively with the COX-2 target (PTGS2), in vitro COX-2 enzyme inhibition assays were performed to verify the anti-inflammatory potential of the January leaf samples. The results showed that the extract exhibited potent inhibitory activity with an IC_50_ of 16.41 μg/mL (95% CI: 14.28–18.54), demonstrating a clear dose-dependent relationship. At a concentration of 60 μg/mL, the extract achieved a COX-2 inhibition rate of 79.43%. The positive control, Celecoxib, confirmed the reliability of the assay system and further supported the anti-inflammatory potential of the *S. suberectus* leaf extract (Figure 6D).

## 3. Discussion

### 3.1. Metabolic Profiling and Seasonal Dynamics of S. suberectus Leaves

A total of 6750 metabolites were identified in *S. suberectus* leaves, with amino acids and their derivatives (26.74%) and organic acids (15.33%) being the most abundant classes. Secondary metabolites, including benzenoids, flavonoids and phenolic acids, accounted for 27.98% of the total metabolome. Amino acids, the most diverse class, participate in various physiological functions, including neurotransmission, immune regulation, and energy metabolism, and have demonstrated potential for development as functional foods, nutritional fortifiers, and feed additives [31,32,33]. Previous studies have shown that legumes tend to accumulate amino acids more readily than other plant families, exhibiting distinct family-specific metabolic accumulation trends [34], which is consistent with our findings in *S. suberectus* leaves. Organic acids, the second most abundant class, are commonly used as preservatives, acidity regulators, and antioxidants, showing promising application potential [35]. In addition, *S. suberectus* leaves contain substantial quantities of plant secondary metabolites, especially flavonoids and phenolic acids, which are widely used in the development of functional beverages, antioxidant health products, and botanical skincare formulations [36,37,38]. These findings provide a solid data foundation and scientific rationale for the integrated utilization of *S. suberectus* leaves in functional food fortification, health product development, and animal nutritional interventions.

The metabolic profile of *S. suberectus* leaves can be divided into three major phases: winter–spring (G1), early summer–autumn (G2), and late summer–autumn (G3). The G1 phase was enriched in functional secondary metabolites, particularly isoflavonoids and flavonoids, which enhance stress resistance and display a typical defensive metabolic pattern. This observation aligns with the well-established concept that plants under abiotic stress (such as low temperature) activate secondary metabolic pathways to produce protective compounds [39]. The G2 phase was dominated by amino acid metabolism and carbon utilization, which are associated with structural maintenance and signal regulation, supporting growth and proliferation. In contrast, the G3 phase was characterized by active primary metabolism, while several key pathways were downregulated, indicating a gradual decline in overall metabolic activity. Collectively, *S. suberectus* leaves undergo stage-specific metabolic modulation to optimize resource allocation and adapt to seasonal environmental changes. This strategy is analogous to that observed in *Zygophyllum dumosum*, which enhances resistance via secondary metabolism during drought and cold seasons [40], and in date palm leaves, which increase amino acid levels under hot conditions to promote photosynthesis and regulate the tricarboxylic acid (TCA) cycle [41].

### 3.2. Selection of Inflammation-Related Pathways and Identification of Anti-Inflammatory Metabolites

In this study, 14 representative inflammation-related pathways were selected from the enriched pathways of differential metabolites in pairwise comparisons among the G1, G2, and G3 stages. The selection was based primarily on anti-inflammatory metabolites enriched in *S. suberectus* leaves and their reported mechanisms of action. These anti-inflammatory pathways were prioritized due to the pivotal roles played by multiple classes of metabolites in inflammation regulation. Specifically, phenylpropanoids, flavonoids, and isoflavonoids exert significant anti-inflammatory and antioxidant effects by modulating inflammatory signaling pathways such as MAPK [42,43,44]. Metabolites from amino acid metabolism, including tryptophan, tyrosine, and arginine, participate in immune regulation and inflammatory responses through the production of bioactive molecules such as nitric oxide (NO) and catecholamines [45,46,47]. Additionally, plant hormones, sesquiterpenes, triterpenoids, and alkaloids are widely involved in anti-inflammatory processes and are important candidate sources for natural anti-inflammatory agents [48,49,50]. Anthocyanins, as common secondary metabolites, have demonstrated considerable anti-inflammatory potential in various in vivo and in vitro models as well as clinical studies [51]. Moreover, fundamental pathways such as carbon metabolism and phenylalanine metabolism not only provide precursors for the synthesis of these active compounds, but their representative intermediates (e.g., citric acid, α-ketoglutaric acid, and phenylalanine) also exhibit immunoregulatory and anti-inflammatory activities [52,53,54,55]. Therefore, the selection of key anti-inflammatory pathways associated with these metabolites facilitates a deeper understanding of the functional metabolic basis and potential anti-inflammatory mechanisms of *S. suberectus* leaves.

Network pharmacology analysis further demonstrated that the anti-inflammatory effects of *S. suberectus* leaves are characterized by a “multi-component, multitarget, and multi-pathway” synergistic regulation pattern. Based on shared enrichment across pathways, 83 putative anti-inflammatory metabolites were identified, mainly comprising phenolic acids (20.5%), flavonoids (19.3%), and organic acids (18.1%). Ten core bioactive compounds were subsequently screened, including flavonoids such as naringenin, apigenin, luteolin, quercetin, genistein, 2′-hydroxygenistein, coumestrol, and pelargonidin; the lignan (+)-secoisolariciresinol; and the alanine derivative N-acetyl-L-phenylalanine. These findings suggest that flavonoids are the predominant contributors to the anti-inflammatory potential of *S. suberectus* leaves, highlighting their potential as a rich natural source of flavonoids with promising applications in functional foods and plant-derived health products. For instance, pelargonidin has been shown to alleviate inflammation by inhibiting the NF-κB signaling pathway [49] and improving intestinal barrier integrity and promoting nutrient absorption [50], demonstrating its potential as a natural anti-inflammatory functional food ingredient. Apigenin inhibits IRAK4, thereby blocking Toll-like receptor (TLR)-mediated inflammatory signaling and effectively mitigating inflammatory responses [51]. Luteolin and quercetin have been reported to suppress pro-inflammatory cytokine production through modulation of the MAPK and PI3K-Akt pathways [56,57].

### 3.3. Key Anti-Inflammatory Targets and Pathway Enrichment Analysis

In our study, key potential anti-inflammatory targets were also identified, including PTGS2, GAPDH, and TNF. PTGS2 (cyclooxygenase-2), a key enzyme in prostaglandin E2 (PGE2) biosynthesis, plays a crucial role in the pathogenesis of chronic inflammation and is a validated therapeutic target [52]. The strong binding affinity between pelargonidin and PTGS2 (binding energy: −11.72 kcal/mol) observed in our molecular docking analysis suggests that *S. suberectus* leaf flavonoids may exert anti-inflammatory effects through direct inhibition of this enzyme. GAPDH, beyond its well-known metabolic functions in glycolysis, has recently been recognized as a regulator of inflammatory signaling in macrophages through malonylation modifications [53]. TNF acts as a central driver of inflammatory cascades by activating cell death pathways and promoting the expression of other pro-inflammatory cytokines [54]. These targets represent the possible mechanisms underlying the multitarget anti-inflammatory effects of *S. suberectus* leaves. Notably, AKT1, SRC, STAT3, and PTGS2 identified in our study have been previously reported as core antioxidant and anti-inflammatory targets in integrated metabolomics and network pharmacology studies on wild jujube fruits and other medicinal plants [26,27], further validating the reliability of our screening approach.

Gene Ontology (GO) analysis indicated that these candidate anti-inflammatory targets are primarily involved in immune regulation, localized in membrane lipid rafts and receptor complexes, and possess kinase or protein-modifying functions. KEGG pathway analysis further revealed their involvement in classical inflammation-related pathways such as MAPK and PI3K-Akt. The PI3K-Akt signaling pathway, in particular, has been identified as a central regulator of antioxidant and anti-inflammatory responses in multiple network pharmacology studies [26,27,28]. The enrichment of these pathways suggests that *S. suberectus* leaf metabolites may exert anti-inflammatory effects via upstream regulatory mechanisms, potentially through the modulation of receptor-initiated signaling cascades. Molecular docking results predicted that the core compounds could stably bind to key targets, with 71% of docking pairs exhibiting binding energies lower than −7.0 kcal/mol, further supporting their potential as natural anti-inflammatory agents.

### 3.4. Seasonal Accumulation Patterns and Optimal Harvest Time

Considering the seasonal variations in metabolite accumulation, January and March appear to be the optimal harvesting periods for *S. suberectus* leaves as potential medicinal resources. During these stages, key anti-inflammatory compounds such as pelargonidin, naringenin, and luteolin are significantly accumulated, indicating strong anti-inflammatory potential. This observation is consistent with studies on other medicinal plants, where seasonal factors significantly influence the accumulation of bioactive compounds. For example, seasonal differences markedly influence the anti-inflammatory and antiproliferative activities of *Bursera microphylla* extracts, with leaf and stem extracts collected in spring exhibiting enhanced bioactivity and pronounced anti-inflammatory effects [58]. Similarly, the content of polyphenol in *Ginkgo biloba* leaves peaks in autumn, correlating with optimal harvest timing for medicinal use [59]. These findings underscore the importance of considering harvest time as a critical variable in the quality control and standardization of plant-derived anti-inflammatory products.

### 3.5. In Vitro Validation: Antioxidant and Anti-Inflammatory Activities

Inflammation and oxidative stress are closely interrelated physiological processes that mutually promote each other, with oxidative stress playing a pivotal role in both the initiation and progression of inflammation [60]. In this study, the leaf extract from the January sample of *S. suberectus* exhibited notable in vitro cell-free antioxidant capacity, with a DPPH radical scavenging rate of 82.25% at 0.33 mg/mL and an ABTS scavenging rate of 81.09% at 0.14 mg/mL. Although these values were slightly lower than those of the vitamin C control, they still indicate considerable antioxidant potential. Antioxidants can scavenge excessive reactive oxygen species (ROS), alleviate oxidative damage, and suppress the expression of pro-inflammatory mediators, thereby contributing to the modulation of inflammatory responses [61]. The radical scavenging ability of the *S. suberectus* leaf extract suggests that it may alleviate inflammation by inhibiting oxidative stress.

Cyclooxygenase-2 (COX-2), an inducible enzyme, is significantly upregulated upon stimulation by pro-inflammatory cytokines and mediates the synthesis of prostaglandins, thereby initiating and amplifying the inflammatory cascade [62]. In vitro cell-free enzymatic assays showed that *S. suberectus* leaf extract inhibited COX-2 activity by 79.43% at a concentration of 60 μg/mL in a dose-dependent manner, providing preliminary evidence for its anti-inflammatory efficacy potential. This COX-2 inhibitory activity is comparable to that reported for other flavonoid-rich plant extracts [21] and corroborates our molecular docking predictions, which identified strong binding affinities between *S. suberectus* flavonoids and the PTGS2 target. As the extract used in this study was a crude, unpurified preparation with complex composition, the observed activity reflects the overall anti-inflammatory potential rather than the effects of individual constituents. This synergistic effect of multiple components is consistent with the fundamental principle of network pharmacology, where multiple compounds interact with multiple targets to produce a holistic therapeutic outcome [26,27].

### 3.6. Limitations and Future Perspectives

It is important to acknowledge the limitations of the current study. First, the network pharmacology and molecular docking employed are in silico predictive tools; thus, the involvement of targets such as MAPK, TNF, and PI3K-Akt represents possible mechanisms of action that require further experimental confirmation. Second, our untargeted metabolomics approach provides relative abundance data rather than the absolute concentrations essential for dose-dependent pharmacological evaluation. Third, the current biological validation relied exclusively on cell-free biochemical assays (DPPH, ABTS, and COX-2 inhibition), which are insufficient to fully demonstrate biological efficacy in a physiologically relevant context.

Therefore, future studies will focus on the following aspects: (1) assessing anti-inflammatory effects in macrophage models (e.g., LPS-stimulated RAW264.7 cells) by measuring NO, IL-6, TNF-α, and NF-κB/MAPK pathways, followed by pharmacokinetic, bioavailability, and safety studies using acute/chronic inflammation animal models; (2) validating purified individual compounds (e.g., pelargonidin, apigenin, and luteolin) to elucidate structure–activity relationships, identify the most potent candidates for functional food development, and perform absolute quantification of key bioactives via HPLC-MS/MS for quality control; and (3) investigating the synergistic interactions among multiple flavonoids and other metabolites to better understand the “multi-component, multitarget” anti-inflammatory mechanisms.

Additionally, it is important to acknowledge that these recommendations are based on a single growth cycle and specific local conditions. To ensure the stability of these metabolic patterns, future studies spanning multiple years and diverse regions, along with assays on purified bioactive compounds, are required to validate the standardized application of these findings.

## 4. Materials and Methods

### 4.1. Materials

Leaf samples of *S. suberectus* were collected from six-year-old cultivated plants at the state-owned Qipo Forest Farm in Nanning, Guangxi, China (108°12′ E, 22°42′ N). All plants were propagated via stem cuttings, with an average stem diameter of around 8 cm. Sampling was conducted in January, March, May, July, September, and November of 2023. At each sampling time point, a total of 15 healthy, pest-free plants were randomly selected and divided into three groups of five plants. Each group of five plants was treated as a single biological replicate. For each plant, mature leaves were collected from the upper, middle, and lower canopy levels, as well as from the four cardinal directions (east, south, west, and north). The leaves from the five plants within each group were then pooled and thoroughly mixed to create one composite sample. This experimental design, using three biological replicates, is a widely accepted standard in metabolomics studies to ensure statistical robustness. To minimize potential batch effects, the order of sample collection in the field and subsequent processing in the laboratory was fully randomized. The plants were cultivated in a uniform plot under consistent management practices at the Qipo Forest Farm. To minimize the influence of diurnal environmental fluctuations, all leaf samples were collected between 9:00 and 11:00 a.m. The leaf samples were flash-frozen in liquid nitrogen and stored at −80 °C until further processing and metabolomic analysis.

HPLC-grade methanol (CAS: 67-56-1) and acetonitrile (CAS: 75-05-8) were purchased from Merck (Darmstadt, Germany). HPLC-grade acetic acid (CAS: 64-19-7) was obtained from Ron Reagent (Shanghai, China). HPLC-grade formic acid (CAS: 64-18-6), ammonium formate (CAS: 540-69-2), and ammonia solution (CAS: 1336-21-6) were purchased from Aladdin Reagent Co., Ltd. (Shanghai, China).

### 4.2. Metabolite Profiling

#### 4.2.1. Sample Preparation and Extraction

Frozen *S. suberectus* leaf samples were retrieved from storage at −80 °C and freeze-dried for 63 h under vacuum using a lyophilizer (Scientz-100F, Ningbo Xinzhi, Ningbo, China). The dried samples were then ground into a fine powder using a tissue grinder (M400, Retsch, Haan, Germany) at 30 Hz for one and a half minutes. An aliquot of 50 mg of the powdered sample was extracted with 1200 μL of a pre-cooled (−20 °C) solution of 70% methanol and water containing internal standards. The internal standard extraction solution was prepared by first dissolving 1 mg of the standard substance in 1 mL of a 70% methanol and water solution to obtain a stock solution of 1000 μg/mL. This was then diluted to 250 μg/mL using a 70% methanol solution to prepare the working solution. During extraction, the mixture was vortexed for 30 s every 30 min, a total of six times. The samples were then centrifuged at 12,000× *g* rpm for three minutes, and the resulting supernatant was filtered through a 0.22 µm microporous membrane. The resulting filtrate was transferred into autosampler vials for subsequent UPLC-MS/MS analysis.

#### 4.2.2. UPLC-MS/MS Metabolite Analysis

Metabolomic profiling was performed using a TripleTOF 6600 mass spectrometry platform (SCIEX, Marlborough, MA, USA) with a Waters ACQUITY Premier HSS T3 column (1.8 μm, 2.1 mm × 100 mm). Separation was conducted at 40 °C with a flow rate of 0.4 mL/min. The injection order of all samples was fully randomized to avoid batch effects. Data were acquired in Data-Dependent Acquisition (DDA) mode via an electrospray ionization (ESI) source. The parameters were: IonSpray Voltage, +5000 V/−4000 V; Source Temperature, 550 °C/450 °C; Ion Source Gas 1 and 2, 50 and 60 psi; and Curtain Gas, 35 psi. The MS1 and MS2 ranges were 50–1000 Da and 25–1000 Da, respectively. For MS/MS fragmentation, the top 18 candidate ions per cycle were selected with a collision energy of 30 ± 15 eV. Raw data were converted to mzXML using ProteoWizard and processed with the XCMS (v3.15.0) package. Blank samples (pure solvent) were injected to monitor carryover and for background subtraction. Missing values were imputed using KNN (v1.56.0), and peak areas were normalized via SVR. Metabolite annotation relied on MWDB V2.0 and public databases. To monitor system stability, pooled QC samples (prepared by mixing equal aliquots of all experimental samples) were injected every ten runs. Only metabolites with a CV < 0.5 in QC samples and an identification score ≥ 0.5 were retained. The high stability of the analysis was confirmed by the tight clustering of QC samples in the PCA score plot and minimal TIC fluctuations (Figure S7). After data preprocessing, multivariate statistical analyses, including principal component analysis (PCA) and partial least squares discriminant analysis (PLS-DA), were performed. DAMs were identified based on the following criteria: variable importance in projection (VIP) > 1.0, *p* < 0.05, and fold change ≥ 2 or ≤0.5. The identified DAMs were annotated to metabolic pathways using the Kyoto Encyclopedia of Genes and Genomes (KEGG) database (Release 2024.02). To evaluate the overall metabolic shift within each pathway, the Differential Abundance (DA) score was calculated using the formula DA Score = (N_up_ − N_down_)/N_total_, where N_up_ and N_down_ represent the number of significantly upregulated and downregulated metabolites in a specific pathway, respectively, and N_total_ is the total number of metabolites annotated in that pathway. A DA score of 1.0 indicates that all metabolites in the pathway were upregulated, while −1.0 indicates all were downregulated. The background list for enrichment analysis consisted of all metabolites successfully annotated in this study.

### 4.3. Network Pharmacology

#### 4.3.1. Screening of Potential Anti-Inflammatory Metabolites and Targets

The canonical SMILES structures of the candidate metabolites were retrieved from the PubChem database (https://pubchem.ncbi.nlm.nih.gov). These structures were then submitted to the SwissADME platform (http://www.swissadme.ch, accessed on 15 February 2024) to evaluate their pharmacokinetic properties and drug-likeness. Metabolites were considered as potential active compounds if they exhibited high gastrointestinal (GI) absorption and satisfied at least two “Yes” criteria in the drug-likeness filters. The potential targets of the selected active compounds were then predicted using the SwissTargetPrediction platform (http://www.swisstargetprediction.ch, 2019 version, restricted to Homo sapiens, accessed on 15 February 2024). To ensure reliability, only predicted targets with a probability greater than zero were retained. The resulting targets were then merged and deduplicated. To identify inflammation-related disease targets, the keyword “inflammation” was used to search the OMIM (https://omim.org, accessed on 15 February 2024), DisGeNET (v24.0, https://www.disgenet.org, accessed in February 2024), and GeneCards (https://www.genecards.org, accessed on 15 February 2024) databases to identify inflammation-related disease targets (v3.0, restricted to Homo sapiens, accessed on 15 February 2024). These three sources were then combined and deduplicated to create a comprehensive set of inflammation-related disease targets. This set was used as the basis for subsequent intersection analysis with the predicted compound targets.

#### 4.3.2. Construction of the “Ingredient–Target–Disease” Network of Anti-Inflammatory Effects and the PPI Network of Common Targets

A Venn analysis was performed to identify the intersection between the predicted targets of the active compounds and the targets of inflammation-related diseases, thereby determining the common targets. The active compounds and their core targets were then imported into Cytoscape 3.10.3 to create a “compound–target–disease” network and visualize the connections. Key anti-inflammatory compounds were identified based on node degree in the network. The intersection targets were then uploaded to the STRING database (https://cn.string-db.org/, accessed on 17 February 2024) to create a protein–protein interaction (PPI) network, with the species set to “Homo sapiens” and a confidence score threshold of ≥0.4. Disconnected nodes were hidden from the network to ensure functional connectivity. The resulting PPI network was then imported into Cytoscape 3.9.1 for visualization. The NetworkAnalyzer plugin and CentiScaPe 2.2 were then used to assess the topological parameters of the nodes. Core targets were screened based on the criterion that their degree, closeness centrality, and betweenness centrality were all greater than or equal to the respective median values. These core targets were then used for downstream functional enrichment and mechanistic studies.

#### 4.3.3. GO Functional Enrichment and KEGG Pathway Analysis

Further exploration of the potential biological functions and signaling pathways associated with the common targets was performed using the Metascape platform (v3.5, accessed on 17 February 2024) (https://metascape.org) via Gene Ontology (GO) enrichment analysis and Kyoto Encyclopedia of Genes and Genomes (KEGG) pathway enrichment analysis. GO analysis included three categories: Biological Process (BP), Cellular Component (CC) and Molecular Function (MF). The threshold for significant enrichment was set at *p* < 0.01. Enriched terms and pathways were ranked based on the Rich Factor and the number of associated genes. The top 20 significantly enriched GO terms and KEGG pathways were visualized using bar and bubble plots generated in R software (version 4.4.1), providing an intuitive overview of the enrichment results.

#### 4.3.4. Molecular Docking

Molecular docking analysis was conducted to evaluate the binding affinity and interaction patterns between the core active compounds in *S. suberectus* leaves and their key target proteins (restricted to Homo sapiens). The 2D structures of the core compounds were retrieved from the PubChem database, and geometry optimization and energy minimization were performed using Chem3D 23.1.1 software to obtain stable conformations. The 3D crystal structures of the key target proteins were obtained from the Protein Data Bank (PDB: https://www.rcsb.org, accessed on 20 February 2024). The protein structures were then preprocessed using PyMOL and AutoDockTools 1.5.7 to remove water molecules and original ligands. Polar hydrogen atoms were added to maintain appropriate protonation states at pH 7.0, and Gasteiger charges were calculated. The docking grid boxes, with a standard dimension of 20 × 20 × 20 Å, were centered on the spatial coordinates of the co-crystallized ligands. Grid box parameters for the binding pockets were subsequently defined for execution in AutoDock Vina 1.1.2. Docking was executed using AutoDock Vina 1.1.2 with the aid of Raccoon VS 1.5.7 for parameter configuration. To validate the protocol, original ligands were re-docked into their receptors, achieving RMSD values of <2.0 Å. While a binding energy of −4.25 kcal/mol served as a baseline, interpretation emphasized pairs with stronger affinities (lower than −7.0 kcal/mol). Interaction patterns were visualized using PyMOL 2.6.

### 4.4. In Vitro Antioxidant and Anti-Inflammatory Capabilities

#### 4.4.1. Sample Extraction

A total of 10 g of freeze-dried and ground *S. suberectus* leaf powder was extracted twice by reflux with 95% ethanol at 10- and 8-fold volumes, respectively. The extracts from both extractions were then combined and filtered. The filtrate was then concentrated under reduced pressure until almost dry, followed by freeze-drying to obtain the crude ethanolic extract powder of *S. suberectus* leaves. The dried extract was sealed and stored for subsequent evaluations of its in vitro antioxidant and anti-inflammatory activities.

#### 4.4.2. DPPH (1,1-Diphenyl-2-trinitrophenylhydr-azine) Free Radical Scavenging Assay

The DPPH radical scavenging assay was performed with slight modifications to a previously reported method [20]. Briefly, 1 mg of DPPH powder was dissolved in 24 mL of 95% ethanol, sonicated for five minutes to ensure complete dissolution and stored in the dark. The absorbance at 517 nm (A_0_) was measured using a mixture of 1 mL DPPH solution and 0.5 mL ethanol, adjusted to a value between 0.6 and 1.0. The test sample was dissolved in 95% ethanol at a concentration of 1 mg/mL. Five gradient volumes (100, 200, 300, 400 and 500 μL) of the sample solution were prepared. For the assay, 1.0 mL of DPPH solution was mixed with varying volumes of the sample and ethanol to give a final volume of 1.5 mL. After being incubated in the dark for 30 min, the absorbance at 517 nm (A) was recorded. DPPH radical scavenging activity was calculated using the following equation, with the DPPH solution plus solvent as the control: Scavenging activity (%) = (1 − A/A_0_) × 100.

#### 4.4.3. ABTS (2,2-Azinobis-3-ethylbenzothiazoline 6-sulphonate) Free Radical Scavenging Assay

The ABTS radical scavenging activity was measured using a modified version of a previously reported method [21]. ABTS and potassium persulfate stock solutions were prepared separately, and 0.2 mL of each was mixed together. This mixture was then incubated in the dark at room temperature for 12 h to generate an ABTS radical cation stock solution. This stock solution was then diluted 10–20 times with 95% ethanol to obtain a working solution with an absorbance of 0.70 ± 0.02 at 734 nm. The test sample was dissolved in 95% ethanol at a concentration of 1 mg/mL. A series of gradient sample volumes (e.g., 20, 40, 60, 80 and 100 μL) was prepared. In the assay, 800 µL of the ABTS working solution was mixed with the sample solution and ethanol to give a final volume of 1 mL. The absorbance (A) was measured at 734 nm after incubation. The control group consisted of an ABTS solution and solvent (A_0_). The ABTS radical scavenging activity was calculated using the following equation: Scavenging activity (%) = (1 − A/A_0_) × 100.

#### 4.4.4. In Vitro Screening of COX-2 Inhibitory Activity

The in vitro anti-inflammatory potential of the samples was evaluated using a Cyclooxygenase-2 (COX-2) Inhibitor Screening Kit (Beyotime Biotech Inc., Shanghai, China) based on a fluorescence method. Celecoxib was used as a positive control. Sample solutions at various concentrations were prepared and diluted with COX-2 Assay Buffer according to the kit instructions. The working solution was freshly prepared by mixing the components provided in the kit in the specified ratios. During the assay, the sample solutions, enzyme, and substrate were added sequentially to a black 96-well plate. Four groups were set up: blank control, 100% enzyme activity control, positive inhibitor control, and sample groups. Fluorescence intensity (relative fluorescence units, RFU) was measured using a microplate reader at an excitation wavelength of 560 nm and an emission wavelength of 590 nm. All measurements were performed in triplicate to ensure data reliability. The COX-2 inhibition rate was calculated using the following formula: Inhibition rate (%) = (RFU100% Enzyme Activity Control − RFUSample)/(RFU100% Enzyme Activity Control − RFUBlank Control) × 100%.

## 5. Conclusions

In conclusion, this study establishes a comprehensive metabolomic profile for *S. suberectus* leaves, providing a robust chemical foundation for their future study. Our analysis successfully identified 6750 metabolites and determined that the G1 period (winter and spring) represents an optimal harvest window, characterized by the peak accumulation of potentially bioactive compounds, including a rich array of flavonoids and phenolic acids. Building on this solid metabolomic data, we performed a preliminary in silico screening to identify candidate compounds and predict possible mechanisms of action. This predictive analysis prioritized 10 core metabolites and suggested their potential interaction with key inflammatory targets (e.g., PTGS2, TNF), possibly through the MAPK, TNF, and PI3K-Akt signaling pathways. The possible mechanisms suggested by computational analysis were supported by our preliminary cell-free assays, which demonstrated that G1-stage leaf extracts possess notable antioxidant and COX-2-inhibitory potential. It must be emphasized that this work is exploratory in nature. The computational models and cell-free assays employed herein do not confirm biological mechanisms or establish a definitive anti-inflammatory effect. Rather, their role is to provide a data-driven foundation and a set of prioritized candidate molecules for subsequent, rigorous investigation. Therefore, this study serves as a critical first step, offering a strong scientific rationale and a clear roadmap for the essential in vitro and in vivo validation required to fully unlock the therapeutic potential of *S. suberectus* leaves.

## Data Availability

The original contributions presented in this study are included in the article/Supplementary Material. Further inquiries can be directed to the corresponding author.

## Supplementary Materials

The following supporting information can be downloaded at https://www.mdpi.com/article/10.3390/plants15101509/s1: Figure S1: Categorization of the 6750 metabolites in *S. suberectus* leaves; Figure S2: Quality control (QC) assessment of the metabolomics data; Figure S3: 3D PCA score plot of samples; Figure S4: The score plots of OPLS-DA pairwise comparisons of differential metabolites; Figure S5: Permutation tests (200 times) for the OPLS-DA models; Figure S6. Categorization of the 83 potential anti-inflammatory metabolite categories; Figure S7: Venn diagram of the overlapping targets between the leaf components of *S. suberectus* and inflammation; Figure S8: The PPI network diagram of common targets for the anti-inflammatory effect exerted by *S. suberectus* leaves; Figure S9: Cluster heatmap of anti-inflammatory metabolites in *S. suberectus* leaves at different periods; Table S1: All sample metabolite data; Table S2: Inflammation-linked plant metabolic pathways; Table S3: SwissADME evaluation of candidate metabolites; Table S4: Target prediction results of active metabolites; Table S5: Target collection results of inflammation.
