# Supplementary material for: Seasonal Metabolic Profiling and Anti-Inflammatory Potential of Spatholobus suberectus Leaves Based on Metabolomics and Network Pharmacology
Source: Plants (Basel). 2026 May 15;15(10):1509. doi: 10.3390/plants15101509 (PMC13210576; doi:10.3390/plants15101509)
Supplement: Supplementary file 1 [file plants-15-01509-s001.zip › plants-4226301-supplementary figures.pdf]

Supplementary figures

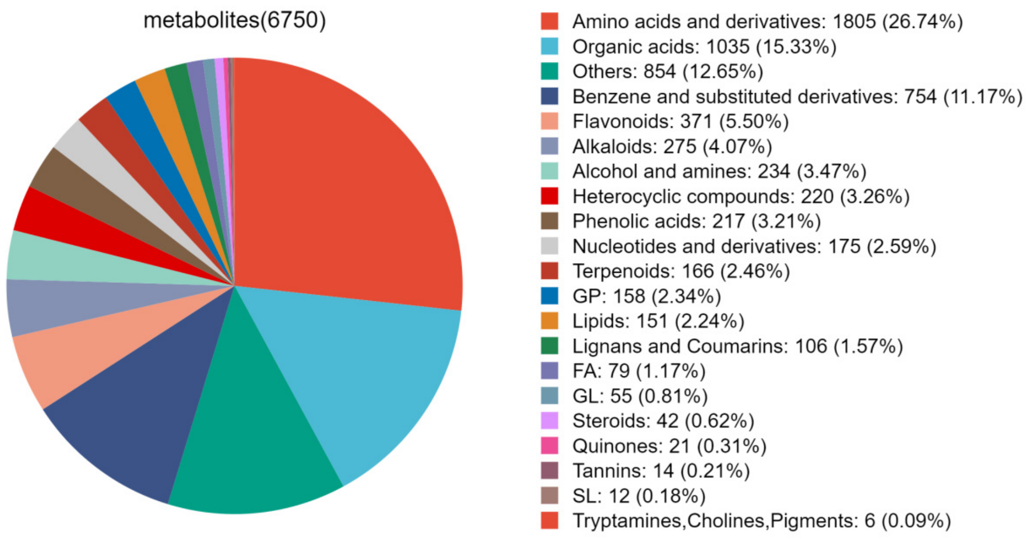

Figure S1. Categorization of the 6750 metabolites of *S. suberectus* Leaves

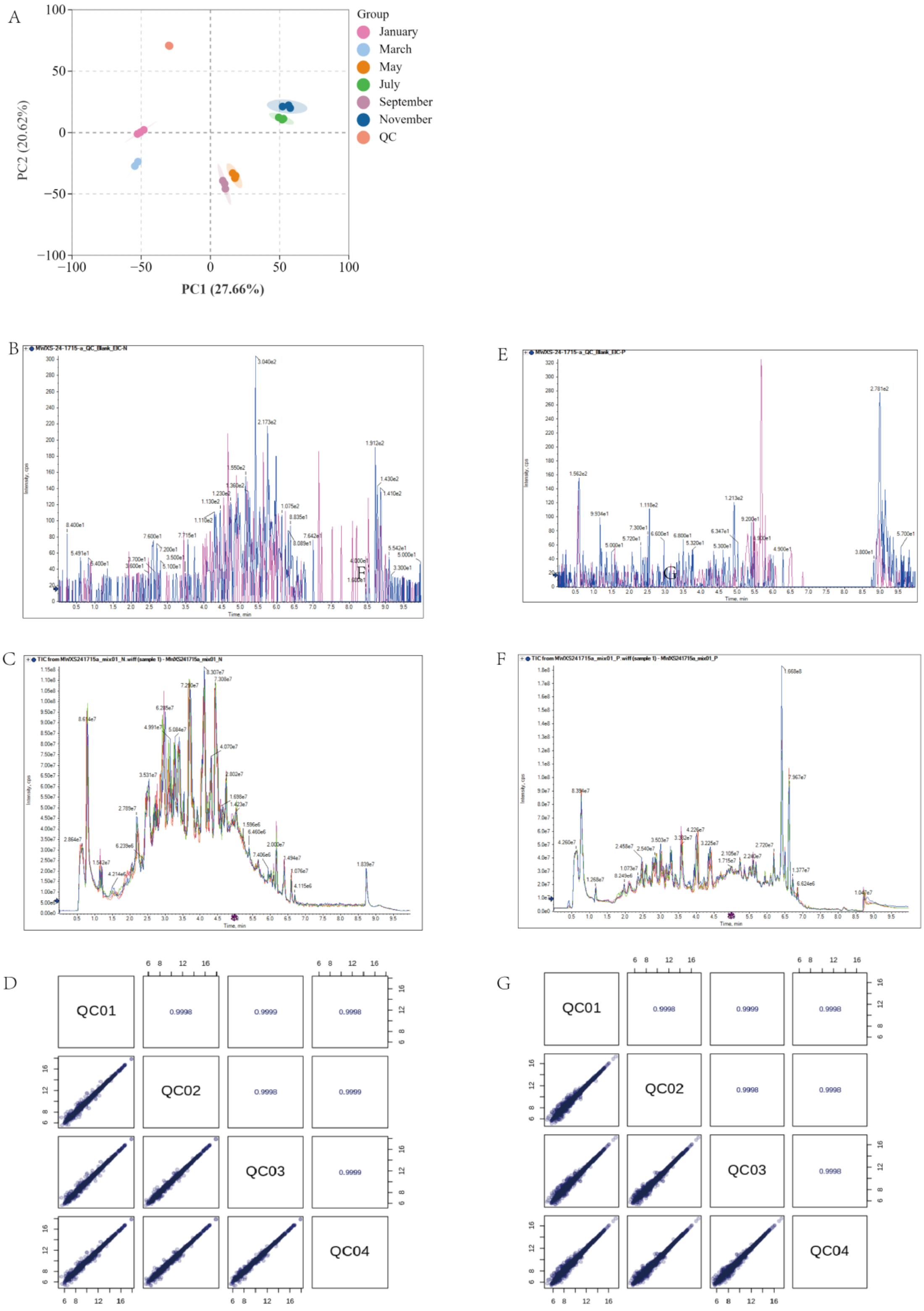

Figure S2. Quality control (QC) assessment of the metabolomics data.(A) PCA score plot of all experimental samples and QC samples. (B – D) QC analysis in negative ion mode: (B) extracted ion chromatograms (EIC), (C) total ion chromatograms (TIC), and (D) Pearson correlation analysis between QC samples. (E – G) QC analysis in positive ion mode: (E) EIC, (F) TIC, and (G) Pearson correlation analysis between QC samples.

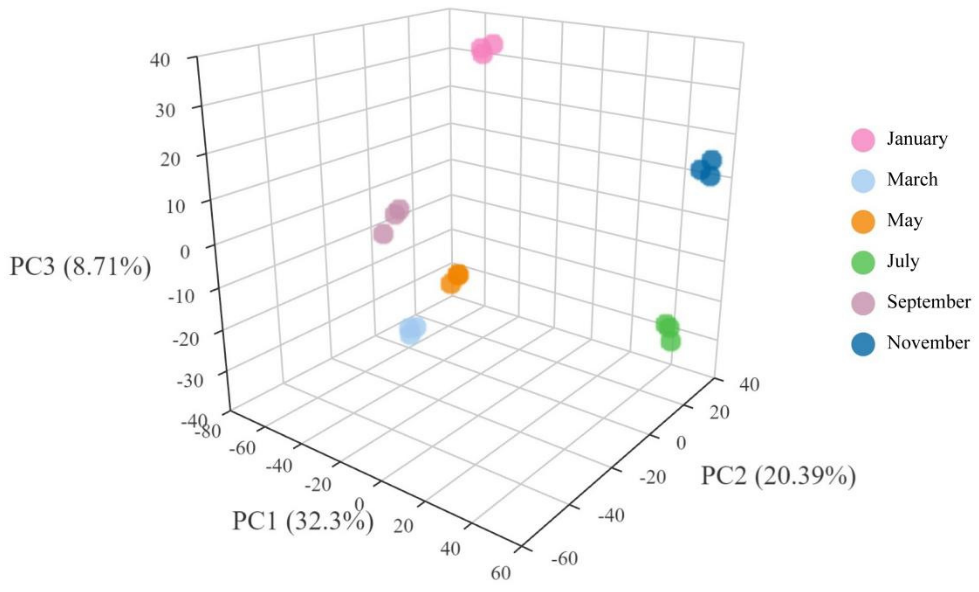

Figure S3. 3D PCA score plot of samples.

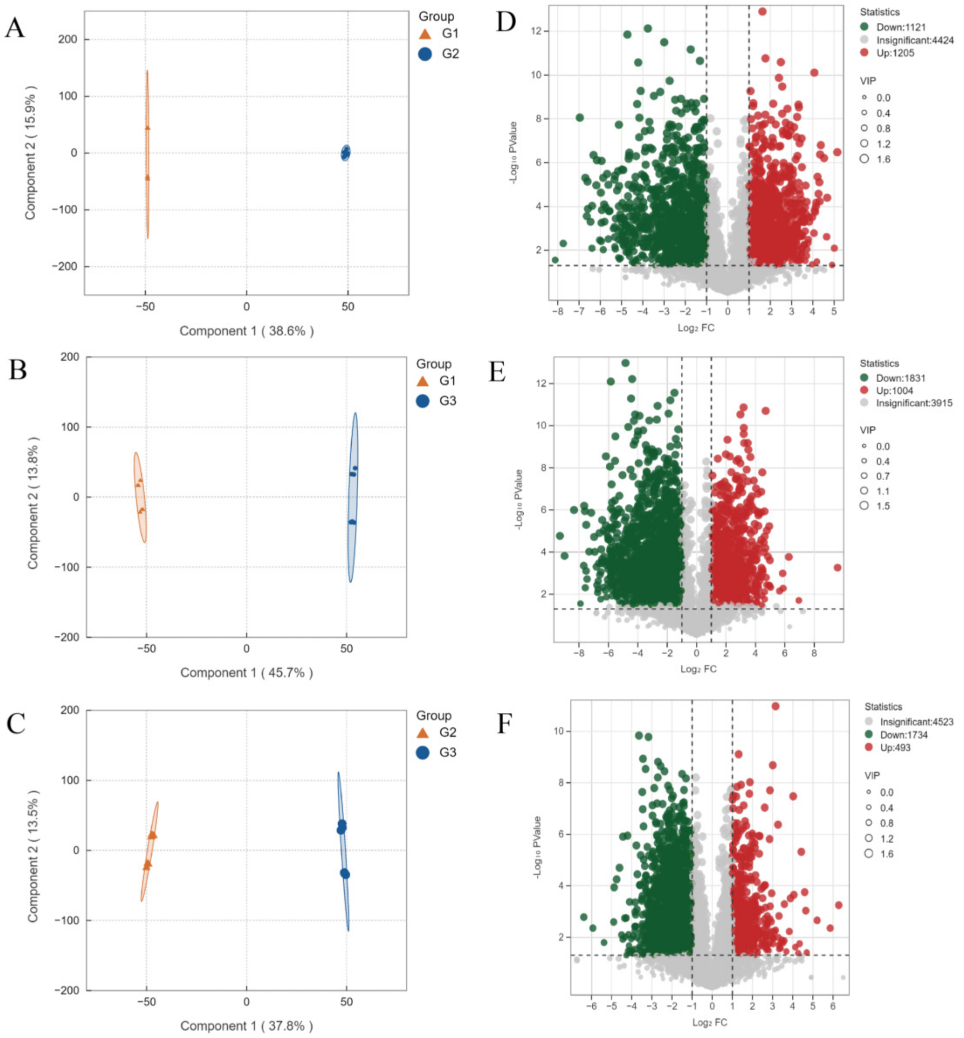

Figure S4. The score plots of OPLS-DA pairwise comparisons of differential metabolites. ((A), G2 vs G1; (B), G3 vs G1; (C), G3 vs G2.. (D-F) Volcano plots showing the differential metabolites expression levels. (D), G2 vs G1; (E), G3 vs G1; (F), G3 vs G2.

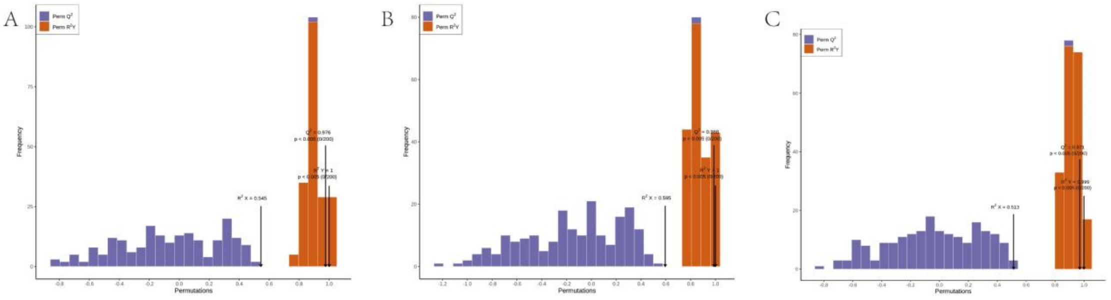

Figure S5. Permutation tests (200 times) for the OPLS-DA models.(A), G2 vs G1; (B), G3 vs G1; (C), G3 vs G2.

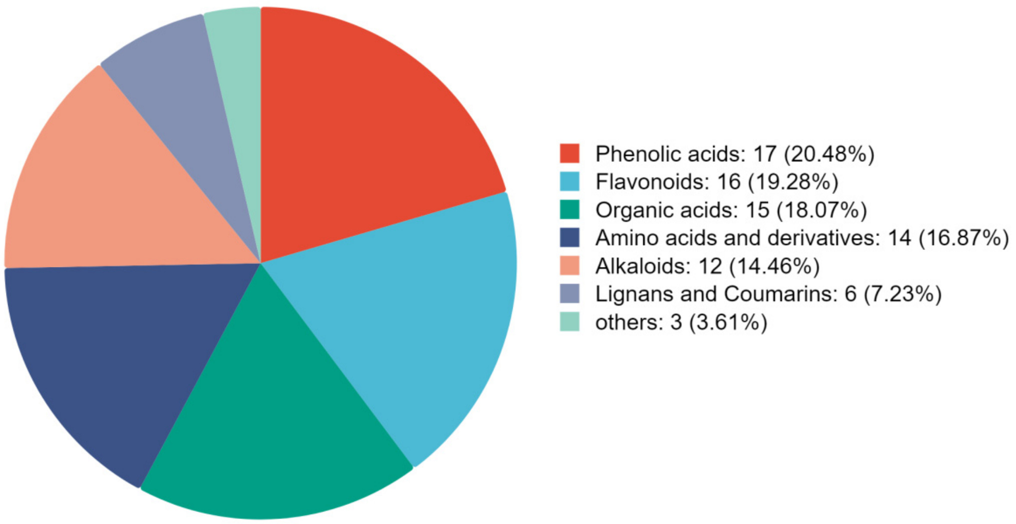

Figure S6. Categorization of the 83 potential anti-inflammatory metabolite categories

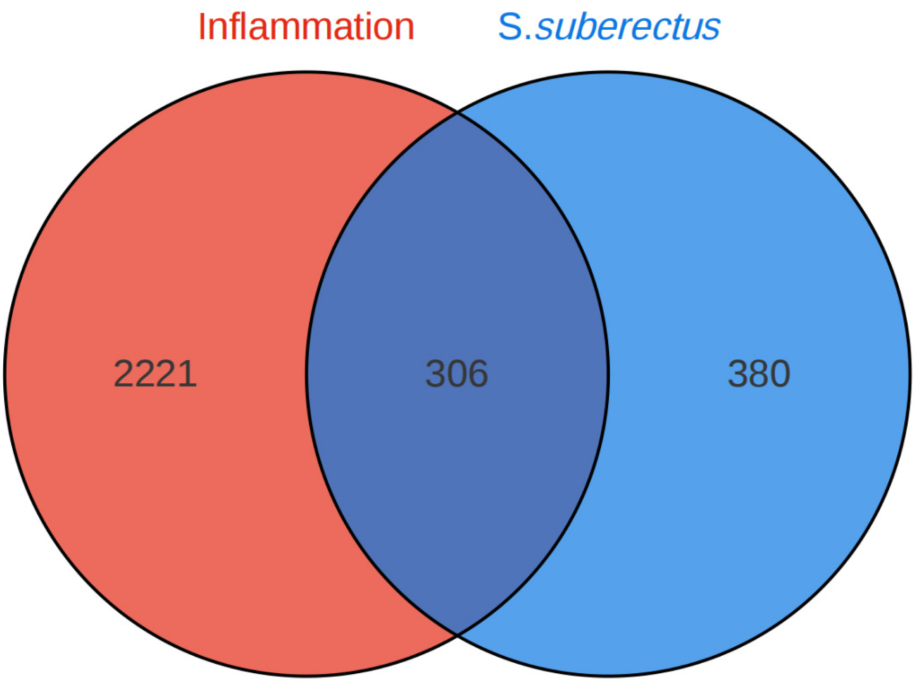

Figure S7. Venn diagram of the overlapping targets between the leaf components of S.*suberectus* and inflammation.

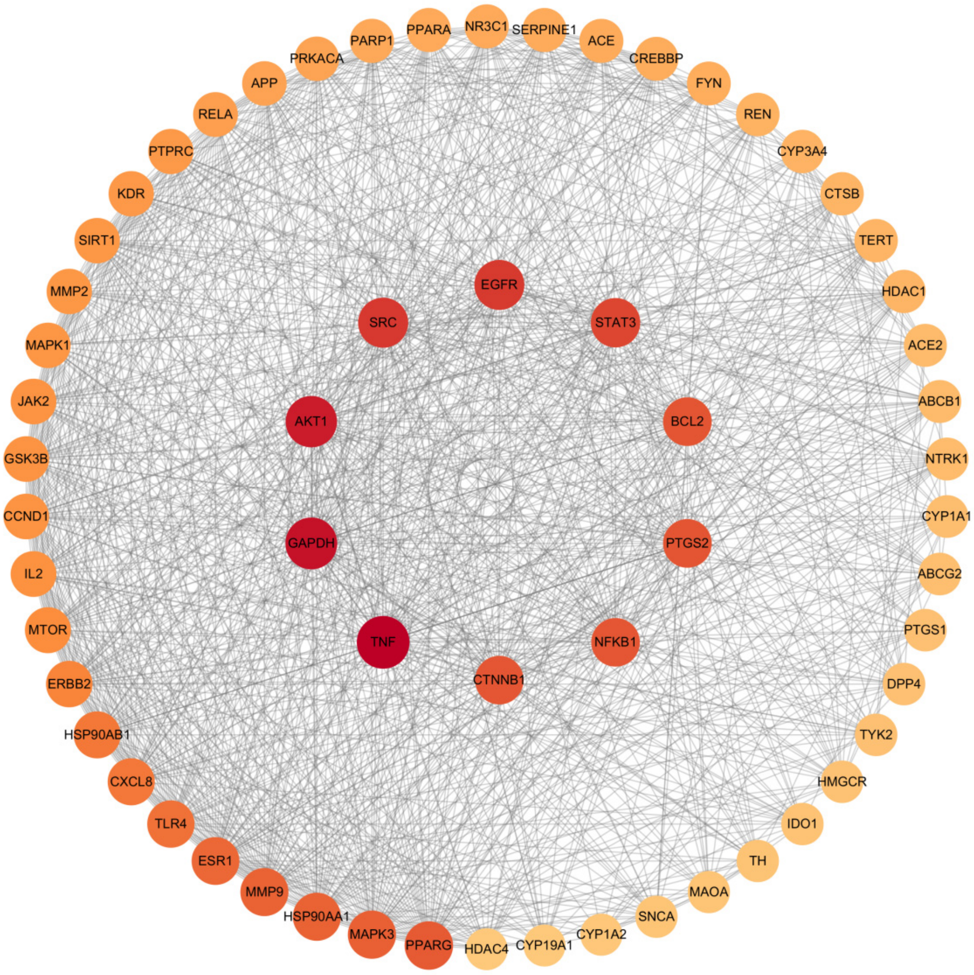

Figure S8. The PPI network diagram of common targets for the anti-inflammatory effect exerted by S.*suberectus* leaves.

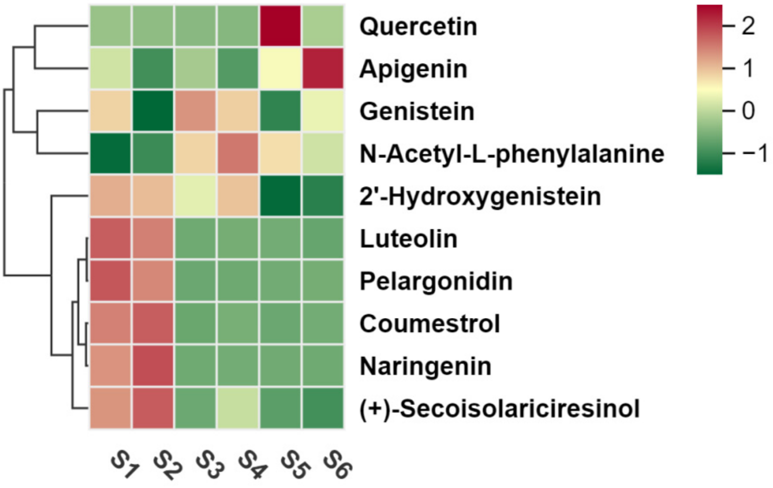

Figure S9.Cluster heatmap of anti-inflammatory metabolites in S. *suberectus* leaves at different periods.
